# Simulation of covariate and concept drift in machine learning hospital admission prediction from emergency triage

**DOI:** 10.1038/s41746-026-02956-0

**Published:** 2026-07-10

**Authors:** Ethan Williams, Toshi Sinha, Matthew Summerscales, Yogesan Kanagasingam

**Affiliations:** 1 School of Medicine, The University of Notre Dame Australia, Fremantle, WA Australia; 2 Emergency Department, St John of God Midland Public and Private Hospitals, Midland, WA Australia

**Keywords:** Computational biology and bioinformatics, Health care, Mathematics and computing, Medical research

## Abstract

Machine learning models that predict hospital admission at triage may support patient flow forecasting, yet the effects of covariate drift, concept drift, and retraining on long-term performance are poorly understood. We developed an Extreme Gradient Boosting (XGBoost) model using deidentified data from all presentations to a metropolitan hospital in Western Australia. Training and validation included 2016 and 2017 presentations (*n* = 133,814), with rolling quarterly testing from 2018 to 2023 (*n* = 455,496). Two adaptive strategies were evaluated: quarterly and half-yearly retraining. Covariate drift was assessed using univariate and multivariable analyses, and reporting adhered to TRIPOD + AI and MINIMAR standards. Substantial drift was observed both between training and testing datasets and across the six-year testing period. The base model achieved a mean AUROC of 0.875 (range 0.844–0.887) and mean daily bed error of 7.42 beds (range 0.47–13.1). Retrained models demonstrated improved discrimination (mean AUROC 0.892 and 0.893) and reduced bed error (4.02 and 4.48 beds per day) for quarterly and half-yearly retraining, respectively, with similar calibration and classification performance. Covariate drift meaningfully degraded calibration but not discrimination metrics over time. Simple retraining improved discrimination and reduced calibration concept drift, underscoring the importance of retraining to address drift for temporal model deployment.

## Introduction

Emergency department (ED) crowding and ramping remain a significant global challenge, associated with delays in patient care, increased morbidity, and reduced healthcare system efficiency, especially in under-resourced hospital settings^1–5^. ED’s are increasingly exploring technological solutions to identify patients requiring admission early in their visit to optimise patient flow and resource allocation^6^.

Recent advances in machine learning (ML) techniques have demonstrated accurate prediction of inpatient admissions using only information available at triage, often with gradient boosted decision trees, random forests and neural networks^7^. Our team’s recent systematic review found that ML models trained exclusively on triage data achieved an area under the receiver operating characteristic (AUROC) from 0.81 to 0.93, with this range considered excellent to outstanding^7,8^. However, little exploration has taken place in this field to assess how feature distributions change over time (covariate drift), nor how these changes impact model performance (concept drift)^7,9–11^. In all fields, it is essential to understand a model’s performance stability over time before prospective real-world implementation, made even more important during times of rapid healthcare environment change, such as during the COVID-19 pandemic^7,12^. While these ML often outperform ED physicians and nurses in accuracy and AUROC^12,13^, bed prediction error is rarely reported^7,9^. Given that bed prediction is the output required for hospital resource planning, it is important to consider how this output changes over time to predict real-world model utility.

The aim of this study was to explore covariate drift, concept drift and concept drift adaptation in a prospective ‘in-silico’ trial, with reporting of clinically relevant bed prediction performance. The objective was to train an XGBoost model on data from a mixed adult-paediatric emergency department from 2016 and 2017 data. The model was tested prospectively on data from 2018 to 2023, reporting covariate drift and model performance quarterly. The secondary aim was to explore if simple concept drift adaptation could improve model performance over time. This study adhered to the Transparent Reporting of a multivariable prediction model for Individual Prognosis Or Diagnosis plus Artificial Intelligence (TRIPOD + AI) guidelines and aligns with the Minimum Information for Medical AI Reporting (MINIMAR) framework for ML research in healthcare^14,15^. This paper aims to contribute both to the technical literature on admission prediction and to guide model development for real-world implementation studies.

## Results

### Demographics

Across the study duration, there were 589,310 presentations, with 51.7% of presentations identifying their sex as female. The training and validation set comprised 133,814 presentations in 2016 and 2017 and 455,496 presentations from 2018 to 2023, forming the quarterly testing sets. The average rate of admission was 30.2% across the eight years, with patients identifying as female having a slightly higher admission rate 30.9%. A higher age and a lower triage category were associated with higher rates of admissions. The most common presenting complaints were “injury limbs”, “abdominal pain”, “other” and “chest pain”. A full list of available demographic data is displayed in Table 1.

**Table 1:** Demographic data of the entire study cohort

Category	Frequency (percentage admitted)			
Age <16 years 16–45 years 45–65 years 65–85 years >85 years	109,323 (14.3%) 227,607(21.8%) 122,342 (33.8%) 100,834 (51.5%) 29,204 (62.7%)			
Triage Category Category 1 Category 2 Category 3 Category 4 Category 5	4,398 (80.1%) 100,396 (51.6%) 224,358 (34.7%) 226,663 (17.5%) 33,495 (4.8%)			
Presentations 2016 2017 2018 2019 2020 2021 2022 2023 Total:	Female	Male	X	Total
	32,648 (30.8%)	31,236 (29.4%)	4 (25%)	63,888 (30.1%)
	35,665 (31.7%)	34,257 (30.9%)	4 (0%)	69,926 (31.3%)
	37,244 (32.6%)	35,592 (30.9%)	1 (0%)	72,837 (31.8%)
	39,844 (29.3%)	38,201 (28.1%)	2 (0%)	78,047 (28.7%)
	38,611 (30.8%)	35,810 (28.6%)	6 (16.7%)	74,427 (29.7%)
	40,394 (29.8%)	36,809 (28.7%)	3 (0%)	77,206 (29.3%)
	39,500 (28.5%)	35,738 (27.7%)	8 (0%)	75,246 (28.1%)
	40,529 (33.5%)	11,700 (31.4%)	10 (30%)	77,733 (32.5%)
	304,435 (30.9%)	284,837 (29.5%)	38 (13%)	589,310 (30.2%)
Presenting Complaints	Injury Limbs: 62,443 presentations (6.8%) Pain Abdomen: 57,501 presentations (38.9%) Other: 43,364 presentations (6.8%) Chest Pain: 41, 452 presentations (41.1%) Pain Limb: 25,795 presentations (16.4%) Shortness of breath: 23,851 presentations (54.9%) Fever: 16,080 presentations (34.85%) Mechanical fall: 10,113 presentations (36.9%)			

### Covariate drift

**Fig. 1:**
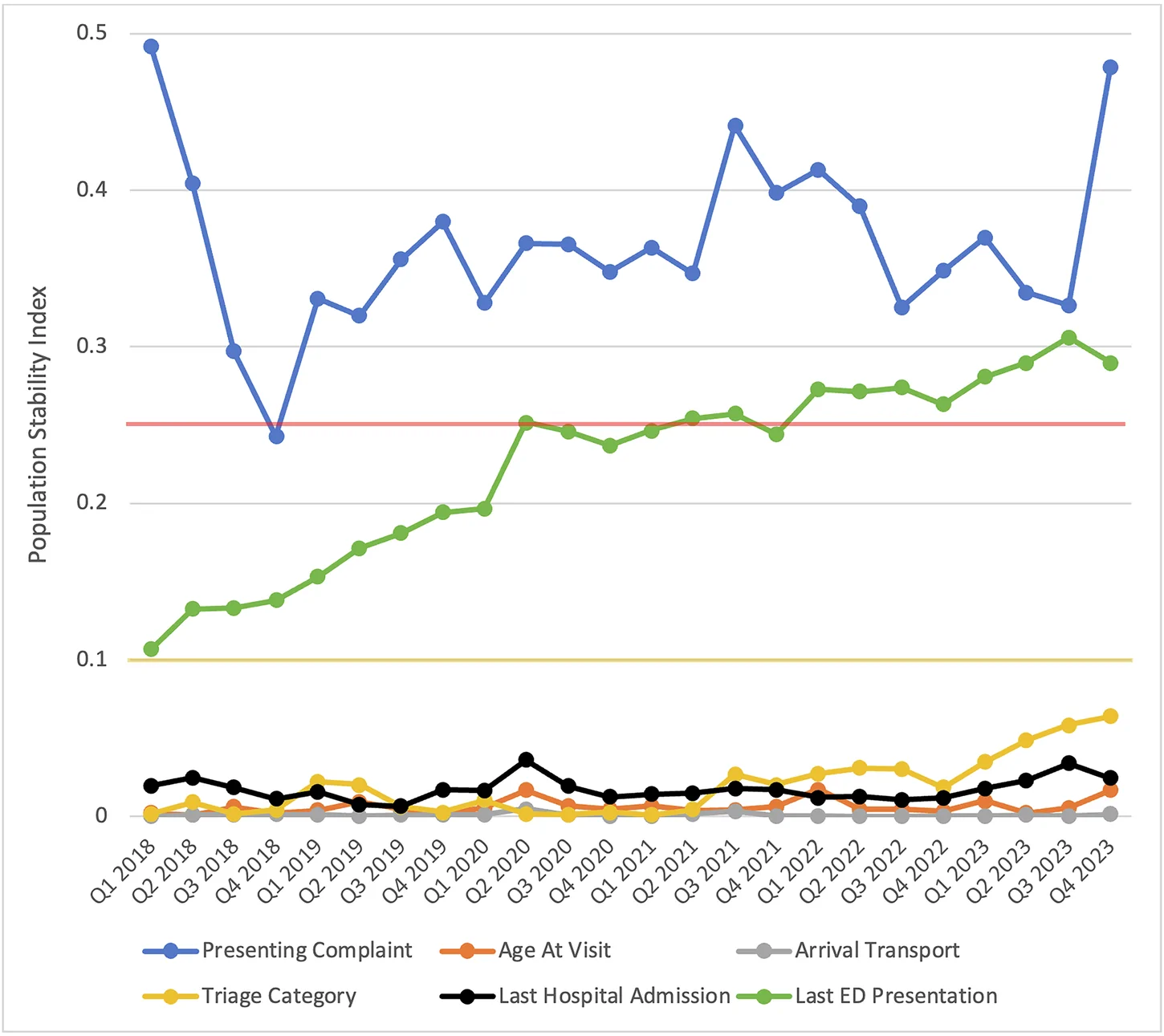
Feature population stability. Population Stability Index for the six most impactful features across the six-year testing window. Key: 0.1–0.25 = moderate instability (yellow threshold); >0.25 = high degree of instability (red threshold).

Presenting Complaint had the highest degree of univariate covariate drift, with a PSI > 0.25 in all but one quarter, seen in Fig. 1. Last ED Presentation had a moderate difference to the test set in 2018 and 2019 (PSI 0.1–0.25), increasing to a high degree of difference for most quarters after 2020. This reflects an increased frequency of representation of patients to the ED over time. Mean age increased by 1.7 years and mean triage score decreasing by 0.2 between the first and last year of the 6-year simulation, though PSI score remained below 0.02 and 0.07, respectively. A slight drift towards higher heart rate and respiratory rates and reduced oxygen saturations was also seen, though PSI scores remained below 0.01. The domain classifier built for multivariate covariate drift detection demonstrated an AUROC of 0.701 when discriminating between presentations in the testing window (2016 and 2017) and the simulation window (2018 to 2023). The PCA reconstruction error also increased over time, indicating increasing divergence from the baseline feature structure. Additional statistics and figures for all feature’s covariate drift are available in the Supplementary Material.

### Base model performance and concept drift

**Fig. 2:**
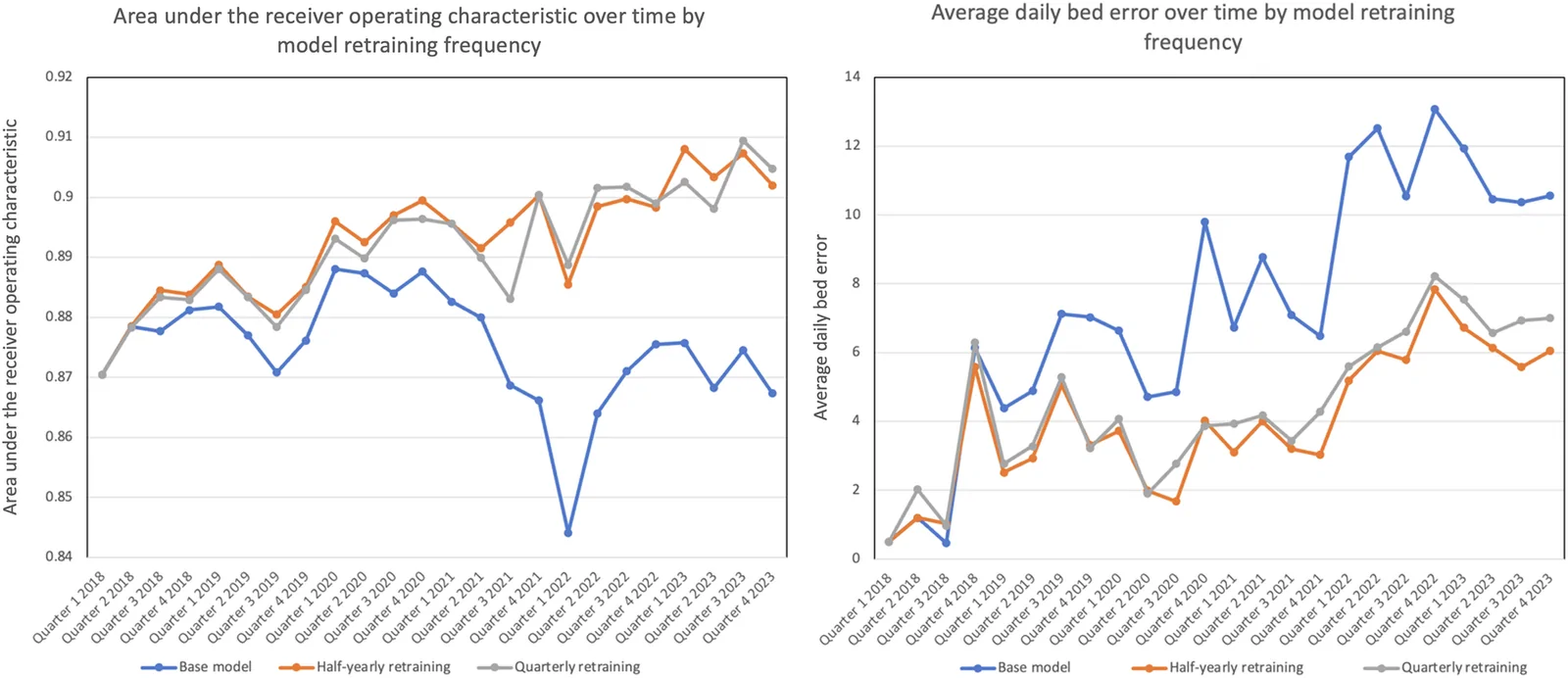
Model performance over time. Area under the receiver operating characteristic and average daily bed error across the six-year simulation by model retraining frequency.

The base model, trained and validated on 2016 and 2017 data, achieved an AUROC of 0.860 in the 2016 to 2017 training set, and a mean AUROC of 0.875 (95th CI 0.869–0.881) during the six-year simulation. Though ADBE rose from 0.04 (95th CI 0.02–0.946) in the training set to a mean of 7.42 (95th CI 5.92– 8.85) in the simulation. There was variable change to other mean outcome measures, with the base model having better bed prediction and ECE results in training, but improved AUROC and accuracy in the simulation, as seen in Table 2. During the simulation, deterioration in performance occurred predominantly between 2020 and 2022, as can be visualised in the plot of AUROC and ADBE in Fig. 2. A full list of quarterly performance metrics for each model over time is included in the Supplementary Appendix.

**Table 2:** Outcome measures for the training set compared to the mean outcomes of the three models over the 6-year prospective simulation

	2016–2017 (training)	2018–2023 (prospective simulation)		
Outcome	Base Model	Base model	Half-yearly retraining	Quarterly retraining
Area Under the Receiver Operating Characteristic	0.860 (0.853–0.868)	0.875 (0.869–0.881)	0.893 (0.887–0.898)	0.892 (0.886–0.897
Accuracy	0.814 (0.807–0.821)	0.830 (0.824–0.836)	0.859 (0.853–0.864)	0.857 (0.852–0.863)
Average daily bed error	0.035 (0.015–0.946)	7.42 (5.92–8.85)	4.02 (3.24–4.78)	4.48 (3.66–5.34)
Sensitivity	0.572 (0.556–0.588)	0.656 (0.647–0.665)	0.645 (0.634–0.658)	0.648 (0.636–0.662)
Specificity	0.929 (0.923–0.934)	0.908 (0.898–0.917)	0.954 (0.950–0.958)	0.951 (0.947–0.955)
Brier score	0.129 (0.125–0.133)	0.119 (0.116–0.123)	0.105 (0.102–0.108)	0.105 (0.103–0.109)
Expected Calibration Error	0.012 (0.008–0.019)	0.047 (0.038–0.055)	0.026 (0.021–0.030)	0.028 (0.024–0.033)

### Performance of concept drift adaptation

The QR and HR model both improved from the base model during the simulation, achieving an AUROC of 0.892 (95th CI 0.886–0.897) and 0.893 (95th CI 0.887–0.898), respectively, and an ADBE of 4.48 (95th CI 3.66–5.34) and 4.02 (95th CI 3.24–4.78), respectively. However, despite gradual improvement in AUROC over the simulation for both models, ADBE also rose over the simulation, reflecting poorer bed prediction over time despite retraining and rising AUROC. Calibration was also sustained with retraining with the HR model attaining a Brier score of 0.105 (95th CI 0.102–0.108) and ECE of 0.026 (95th CI 0.021–0.030) compared to 0.119 (95th CI 0.116–0.123) and 0.047 (95th CI 0.038–0.053) for the base model. When comparing NC-DiD, there was no difference between the QR and HR models in AUROC, ADBE, accuracy, Brier score or ECE with estimates of 0.0009 (95th CI −0.0017 to 0.0034), −0.12 (95th CI −0.71 to 0.46), 0.0019 (95th CI −0.0034 to 0.0073), −0.0009 (95th CI −0.0030 to 0.0012), 0.0015 (95th CI −0.0033 to 0.0002) respectively. Performance of all key metrics over time and an overall calibration curve is included in the Supplementary Materials.

### Feature importance

**Fig. 3:**
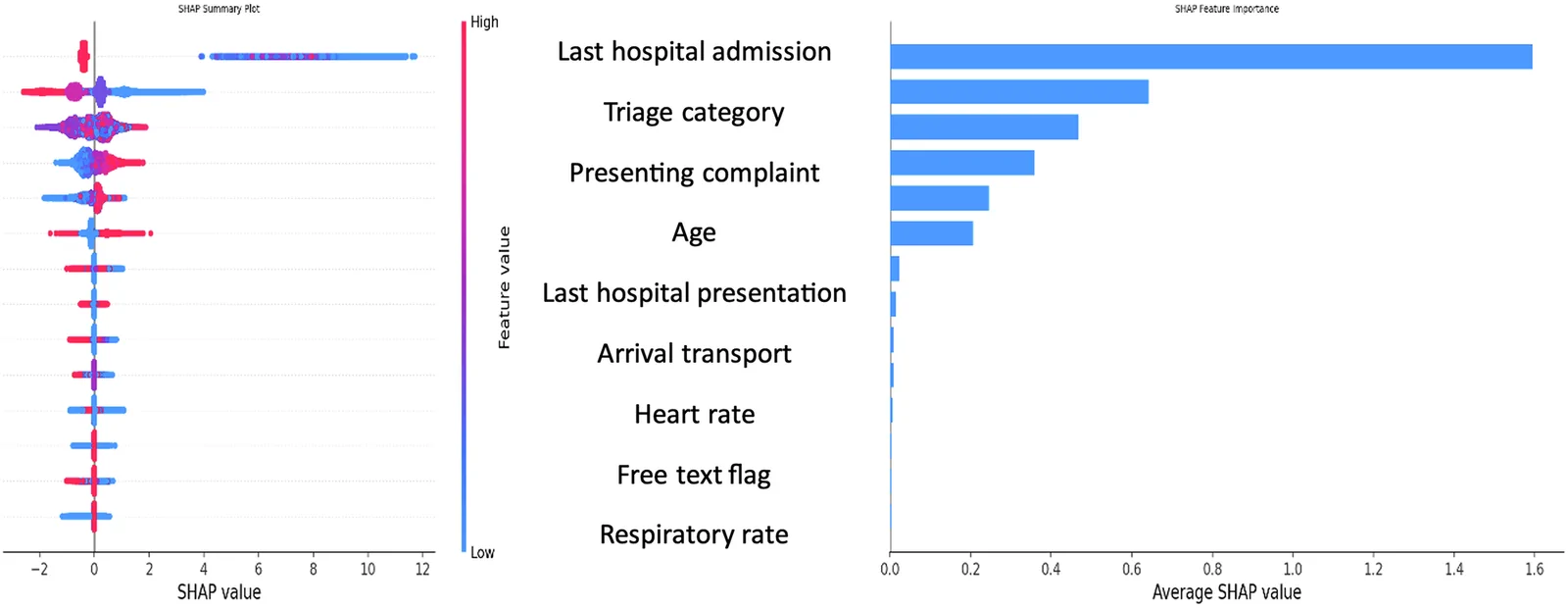
Feature importance. SHAP analysis of pooled feature importance for the quarterly retrained model across the six-year testing period.

The time since hospital admission was the most useful feature identified by the SHAP analysis, with recent admissions increasing the likelihood of another admission. Triage category, presenting complaint, time since last ED presentation, age and mode of arrival were the next five most impactful features. Figure 3 plots how each feature contributes to the model’s prediction, with positive SHAP values predicting admission and negative SHAP values predicting discharge.

## Discussion

While the population in this study has not previously been studied, the demographics and admission rates are comparable to previous studies in the field^7,8,10,16^. The base model’s AUROC of 0.860 during training and 0.875 during testing is in line with the highest performing models when predicting admission from ED triage data^8–10,17,18^. Though, reduction in some performance metrics and the variability of AUROC between 0.844 and 0.887 during the simulation is a novel finding. SHAP analysis is congruent with other high performing models, with triage category, previous admissions, previous ED presentations, mode of arrival and age all commonly reported highly impactful features^8–10^. King et al. is the only other paper to report a bed error metric, reporting a mean absolute bed error of 4.0^9^. While the timescale at which bed error was calculated did vary in this study, the base model initially had comparable accuracy before drift, and the QR and HR models sustained comparable performance across the simulation.

This study provides evidence that within a prospective simulation, an XGBoost model can sustain admission prediction performance over time with retraining. This was observed despite covariate drift across the dataset as PCA reconstruction error over time, indicating increasing divergence from the baseline multivariate feature structure. This was supported by an AUROC of 0.701 for a XGBoost model discriminating presentations in the training set from those in the testing window. Univariate analysis demonstrated drift in all features, though only presenting complaint and time since last ED presentation had moderate to high drift as measured by the PSI. Increased AUROC over time was noted for both retrained models across the six-year period and while the base model sustained a mean AUROC higher than the training set, the value declined in the later years of the simulation. Despite rising AUROC, ADBE also rose during this period for all models, indicating worse bed predictive performance. The gap in AUROC and ADBE between the base model and the retrained models grew over the six years, most notably after the third quarter of 2020. This performance drift is partially explained by the moderate to high PSI score for presenting complaint and time since last ED presentation, which were also the third and fifth most important features in model performance. The demonstrated covariate and concept drift were best assessed over years, with initially similar performance in 2018, with the largest differences in 2022 and 2023. This is supported by rising PCA reconstruction error over time. This indicates that long-term covariate and concept drift may have been unappreciated in previous literature using shorter temporal validation windows, or by focusing on AUROC^9,10^.

The counterintuitive divergence between discrimination and calibration over time is an important finding when considering clinical implementation. While AUROC remained stable or improved, calibration metrics such as ECE and ADBE rose during the testing period without retraining. The worst quarterly deterioration of all metrics was in the first quarter of 2022, coinciding with Western Australia’s first major COVID-19 wave. Although the impacts of COVID-19 were likely attenuated given the high vaccination rates of 94.1% of residents in Western Australia having received at least one dose of COVID-19 vaccination by January 16 2022^19,20^. Given the degree of covariate drift and subsequently concept drift observed during this period in Western Australia, we hypothesise the magnitude may be greater in settings with higher COVID-19 caseloads and mortality rates. Importantly, retraining cycle occurred with all previously available data, so large but recent shifts in presenting or admitting patterns may still be greatly underrepresented in the entire training set. This is an important finding that AUROC may not always reflect the calibration and bed prediction capacity of a model. Further research should explore variable training strategies to combat this calibration drift, exploring variable training windows, or the use of temporally recent validation sets to tune models before testing on the next testing window.

Despite large performance variations between the base model and retrained models, the NC-DiD analysis revealed no difference in performance between the HR and QR models. This may suggest that with half the computational demand, accuracy may be maintained with half-yearly retraining. Given addition frequencies of retraining were not explored, further studies should assess a wider range of retraining intervals, drift detection, retraining techniques and the impact of retraining on different models such as neural networks.

The type of modelling utilised in this study could be operationalised through integration with hospital dashboards or planning tools, offering near real-time forecasts of anticipated inpatient demand based on the current ED population. By pooling admission probabilities to calculate ADBE, rather than identifying individual patients for admission, the model provides aggregate predictions well suited to the needs of bed flow managers tasked with system-level resource allocation. It is important to consider local and model specific requirements beyond population factors when both interpreting model performance and suitability. For example, this study included hospital transfers as admissions due to their local and system-wide resource requirements. However, as seen in the Supplementary Material training data, recording transfers as discharges in training can improve the model’s performance, and could be relevant in scenarios where strictly site-specific inpatient bed prediction is desired. While this study offers a novel in silico simulation of prospective use with quarterly model assessment, the exploration of additional retraining techniques and real-world validation at clinically relevant timescales is required to assess integration performance and impact on patient flow.

Model performance drift is likely to vary between healthcare settings based on population characteristics, healthcare service changes, seasonal changes and unprecedented epidemics and pandemics. This study is limited to a single healthcare environment during a period including a pandemic, though optimal concept drift adaptation is likely to change over time and between healthcare settings. Therefore, extrapolation of exact performance measures to other sites may be imprecise and variable over time. However, this study demonstrates previously unexplored relationships in covariate and concept drift, which likely apply across other healthcare settings given their dynamic nature, though the type and degree of drift may differ. Only simple and infrequent concept drift adaptation methods were explored in this study, due to the limited prior evidence for concept drift adaptation in this field. Exploration of alternative model hyperparameters, model types, retraining windows and techniques to optimise performance over time in a variety of healthcare settings may be warranted. Finally, the ‘in silico’ nature of this prospective simulation study requires real-world validation to confirm whether these predictions can positively assist patient flow managers in optimising resource allocation and improve patient outcomes.

This study demonstrates that an XGBoost model can sustain predictive performance over time with at least half-yearly retraining in this simulated environment. Covariate drift was notable, though even without retraining, AUROC improved over time while calibration metrics had progressive deterioration. Simple retraining optimised model performance over time, with discrimination metrics improving and calibration metrics declining at a slower rate than the base model. Given this discordance, while AUROC remains a core performance metric, calibration metrics and operational measures like ADBE should be explored in future research to give insight into real-world utility. These findings underscore the value of ongoing covariate drift and concept drift monitoring, with shifts in population structure and healthcare delivery practices emerging as critical determinants of model performance over time.

## Methods

### Data collection and ethics

The data was sourced from a single medium sized, non-tertiary, mixed paediatric-adult hospital within the Perth, Western Australia, metropolitan area. All presentations from January 1, 2016, until December 31, 2023, were included. All triage data fields were extracted by the hospital’s data custodian and deidentified before being provided to the research team in accordance with the ethics approval by St John of God Health Care Human Research Ethics Committee (#2018) and ratified by The University of Notre Dame Australia (#2015-081). A waiver of consent was granted for the use of exclusively deidentified data. Clinical trial registration was attained from the Australian New Zealand Clinical Trials Registry before model development (ACTRN12624000615583). The datasets generated and analysed during the current study are not publicly available due to our ethics agreements, but are available from the corresponding author on reasonable request. The model code may also be made available on reasonable request.

### Features and outcome definition

All available features recorded at ED triage were included. Notes, test results and data input at any time point after triage were not retrieved. Features available for each patient were age, sex, suburb, arrival transport, triage category, presenting complaint, last presentation to the ED, last hospital admission and triage nurse free text, which often included observations. The Australasian Triage Scale, developed by The Australasian College for Emergency Medicine, is used by trained triage nurses to determine triage category from one to five, with one being the most urgent^21^. Vital signs were extracted from triage nurse free text, if documented, and 16 free text flags were created, such as “collapse” or “seizure”. The full list of flags and their definitions can be found in the Supplementary Appendix. The outcome variable of admission was defined as any patient admitted to an inpatient hospital ward, including the ED short stay ward. As the hospital was a non-tertiary centre, patients who were transferred to another hospital for assessment or admission were also included as admissions. While counting these patients as discharges or excluding them improved performance in our sensitivity analysis, included in the Supplementary Material, they are included as admissions to reflect the resource demands of the clinical scenario and in alignment with the literature^22–24^. These patients often require an admission within the health system and frequently occupying a bed for multiple hours or days prior to transfer. Patients who left against medical advice or left without review were categorised as discharges, while patients who passed away in the department were excluded from analysis. A patient’s last ED presentation and last hospital admission was set to three years if the fields were blank to avoid computation of a ‘0’ value for patients who had not recently attended hospital. The remaining missing data is natively handled by the XGBoost model. No further data or feature pre-processing was conducted to best simulate the environment of a model running in a real-world setting.

Univariate covariate drift was assessed by initially plotting the quarterly mean values of each individual feature over the testing period in reference to the training set mean. Population stability index (PSI) was used to quantify covariate drift for each feature over time by comparing training and testing feature distributions across predefined bins, with values of 0.1–0.25 indicating moderate drift and values greater than 0.25 indicating substantial drift. For multivariate covariate drift, an XGBoost model was built on the entire dataset to discriminate between presentations in the study’s training window (2016 and 2017) compared to presentations in the testing window (2018–2023). The same feature handling and hyperparameters as the main model were used for this domain classifier. Finally, principal component analysis (PCA) reconstruction error was calculated across the testing window compared to the training window to plot the multivariate covariate drift over time.

### Model development

The XGBoost algorithm was chosen over logistic regression and neural networks after initial testing on the 2016 and 2017 training set. A randomised 80% of the 2016 and 2017 presentations were used in the training, 10% in cross validation group and the remaining 10% were used for model selection testing. XGBoost was the best of the three models tested, aligning with findings in the literature^7,25,26^. These selection results can be found in the Supplementary Appendix. XGBoost grid search was run to select the optimal hyperparameters, again from the 2016 and 2017 dataset, which is temporally separate to the simulation test set. These hyperparameters were held constant across the simulation to avoid additional confounding variables. A learning rate of 0.01, eta of 0.1, maximum number of trees of 1000, max delta step of 10, maximum depth of 10, subsample of 0.9 and fraction of features 0.9 were used. Synthetic Minority Over-sampling Technique (SMOTE) was used to account for class imbalance. SHAP analysis was then extracted from the quarterly retraining model to provide the relative impact of each feature on model performance.

The XGBoost model trained as above was labelled the ‘base model’ and was tested on each successive quarter from 2018 to 2023 in a prospective simulation, without any retraining. Performance measures chosen aligned with other papers currently published, including AUROC, accuracy, sensitivity, specificity, Brier score and expected calibration error (ECE), with the additional measure of average daily bed error (ADBE)^7,9^. ADBE is the difference between the model’s predicted daily admission count and the actual admissions on a given day, averaged out over each quarter. Daily admission count was the sum of individual patient’s predicted probability of admission, with sensitivity, specificity and accuracy calculated with Youden’s index to set the threshold.

### Two further models were created to compare two basic concept drift adaptation variations

1. Quarterly retraining (QR) model: The QR model was made with the same starting hyperparameters as the base model. However, after it was tested on data from the first quarter of 2018, it was then retrained with data from 2016, 2017 and the first quarter of 2018 before testing on the second quarter of 2018. The training set continued to expand each retraining cycle, with no maximum size for the training set. This retraining was repeated quarterly, creating an updating model with testing always occurring with novel temporally separate data.
2. Half-yearly retraining (HR) model: The HR model was retrained in the same way as the QR model, though retraining only occurred on a half-yearly basis, such that the model retrained after being tested on the second and fourth quarter each year. A visualisation of this training methodology can be found in the Supplementary Material.

Quarterly performance measures of these models were plotted against each other for temporal analysis of model performance. Given the QR and HR model would retrain on the same data prior to first and third quarter data testing each year, and only the QR would retrain prior to second and fourth quarter testing, further analysis was performed to assess the impact of more regular retraining. A negative control difference in differences (NC-DiD) analysis was performed between the models, treating quarter one and three as the control group and quarter two and four as the intervention group, aligning with the methodology of other digital health data studies^25^.

## Supplementary information

Supplementary Information

## Data Availability

The datasets generated and analysed during the current study are not publicly available due to our ethics agreements, but are available from the corresponding author on reasonable request. The model code may also be made available on reasonable request.

## Code availability

The rights of the specific patient dataset and code for this project have not been approved for publication or sharing under the current ethics agreement. Any applications for access to this dataset would need to be approved by the data custodian and the St John of God Health Care Human Research Ethics Committee.
