# Supplementary material for: Simulation of covariate and concept drift in machine learning hospital admission prediction from emergency triage
Source: NPJ Digit Med. 2026 Jul 10;9:770. doi: 10.1038/s41746-026-02956-0 (PMC13646290; doi:10.1038/s41746-026-02956-0)
Supplement: Supplementary file 1 [file 41746_2026_2956_MOESM1_ESM.pdf]

Model free text flag definitions:

Flag 1: "fits", "seizure", "status epilepticus"

Flag 2: "collapse", "fall"

Flag 3: "b-fast","bfast"

Flag 4: "anaemia", "anaemic", "anemia"

Flag 5: "neutropaenia", "immunosuppressed", "immunosuppression", "neutropenic", "neutropenia", "immune suppression", "immune suppressed"

Flag 6: "chemo", "chemotherapy", "radiotherapy"

Flag 7: "chest pain", "pain chest"

Flag 8: "hypertension", "htn"

Flag 9: "diabetes", "diabetic", "t2d"

Flag 10: "haematemesis", "gi bleeding", "blood" AND "vomit"

Flag 11: "haematuria"

Flag 12: "pancreatitis"

Flag 13: "AAA", "aortic aneurysm"

Flag 14: "abscess" BUT NOT "pilo"

Flag 15: "sepsis"

Flag 16: "asthma", "copd", "breathless"

Flag 17: "heart failure", "CHF", "stemi", "nstemi"

Flag 18: "overdose","poisoning"

Flag 19: "sent", "ref", "referred", “gp”

Flag 20: "transfer", "expect"

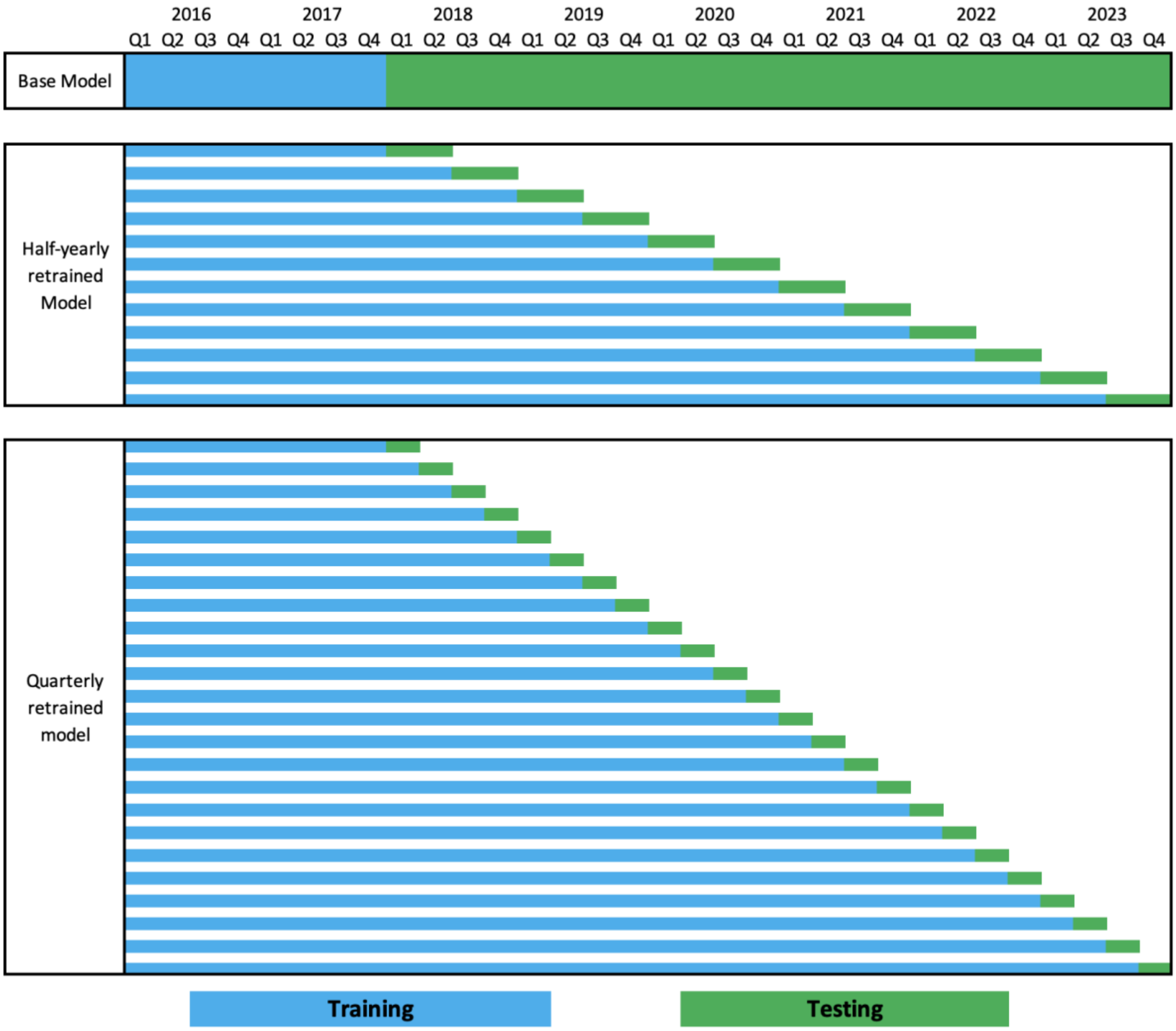

Supplementary Figure 1: Visualisation of the simulation comparing the training and testing frequency and composition of the base model, half-yearly model and the quarterly model.

Base Model (no retraining):

Quarter	Area Under the Receiver Operating Characteristic	Lower 95% confidence interval	Upper 95% confidence interval	Accuracy	Lower 95% confidence interval	Upper 95% confidence interval	Average daily bed error	Sensitivity	Specificity	Brier score	Expected calibration error
2018 1st Quarter	0.870468	0.864654	0.875696	0.821862348	0.81589767	0.827827	0.509851	0.614246	0.927623	0.124328	0.016912
2018 2nd Quarter	0.878449	0.872686	0.884175	0.833438845	0.827628549	0.839249	1.202293	0.612928	0.944503	0.119021	0.01533
2018 3rd Quarter	0.877687	0.871852	0.883338	0.840605686	0.834965526	0.846246	0.467709	0.624717	0.945536	0.116174	0.011219
2018 4th Quarter	0.881218	0.875902	0.88702	0.841518259	0.835975697	0.847061	6.140013	0.645829	0.925862	0.114164	0.033504
2019 1st Quarter	0.881785	0.875811	0.887811	0.8478391	0.842392567	0.853286	4.383183	0.640616	0.938053	0.110996	0.02695
2019 2nd Quarter	0.876997	0.871235	0.882843	0.844978166	0.839528954	0.850427	4.882206	0.629493	0.931854	0.112646	0.03234
2019 3rd Quarter	0.870814	0.863196	0.876832	0.844147278	0.838642136	0.849652	7.120906	0.623478	0.928205	0.112187	0.041471
2019 4th Quarter	0.87613	0.87048	0.88175	0.824060756	0.818400036	0.829721	7.030091	0.636943	0.907	0.120748	0.036807
2020 1st Quarter	0.88804	0.882935	0.893543	0.844716495	0.839283691	0.850149	6.643635	0.655152	0.926013	0.110098	0.035413

2020 2nd Quarter	0.887361	0.881361	0.89323	0.837758314	0.831781027	0.843736	4.711871	0.674347	0.919204	0.113648	0.03055
2020 3rd Quarter	0.884007	0.878138	0.88935	0.841433451	0.835891348	0.846976	4.85982	0.651869	0.925721	0.111077	0.030663
2020 4th Quarter	0.887611	0.882113	0.893446	0.835899015	0.830202784	0.841595	9.794412	0.67511	0.902905	0.113538	0.054882
2021 1st Quarter	0.88261	0.876009	0.888105	0.843941718	0.838255568	0.849628	6.725811	0.65781	0.923049	0.110367	0.039114
2021 2nd Quarter	0.879944	0.873601	0.885174	0.826613423	0.820658484	0.832568	8.778422	0.668613	0.897214	0.117761	0.051452
2021 3rd Quarter	0.868689	0.862218	0.874658	0.82342931	0.817263433	0.829595	7.094254	0.664849	0.899627	0.122311	0.047786
2021 4th Quarter	0.866117	0.859282	0.871759	0.822567195	0.81657009	0.828564	6.478076	0.640454	0.909143	0.122728	0.039606
2022 1st Quarter	0.844059	0.83691	0.850867	0.791950887	0.785024736	0.798877	11.68355	0.654577	0.851675	0.141558	0.0838
2022 2nd Quarter	0.86401	0.856988	0.870793	0.807396871	0.800878626	0.813915	12.52224	0.681948	0.861015	0.13151	0.081047
2022 3rd Quarter	0.870986	0.86401	0.877924	0.832088274	0.826023274	0.838153	10.54646	0.668529	0.90034	0.118122	0.065775
2022 4th Quarter	0.875515	0.869288	0.88134	0.831100333	0.825382743	0.836818	13.07234	0.678134	0.892745	0.118562	0.072118
2023 1st Quarter	0.875756	0.869422	0.882189	0.829288247	0.823199231	0.835377	11.93188	0.684605	0.891368	0.121433	0.074025
2023 2nd Quarter	0.868232	0.862003	0.874373	0.8196583	0.813339756	0.825977	10.46564	0.677191	0.886218	0.128909	0.06696
2023 3rd Quarter	0.874517	0.86789	0.881222	0.820641424	0.814321446	0.826961	10.36597	0.690866	0.88396	0.126425	0.066636
2023 4th Quarter	0.867325	0.860795	0.874066	0.821341899	0.814998787	0.827685	10.56366	0.689984	0.883559	0.127587	0.068615

Half-yearly retraining (half-yearly model):

Quarter	Area Under the Receiver Operating Characteristic	Lower 95% confidence interval	Upper 95% confidence interval	Accuracy	Lower 95% confidence interval	Upper 95% confidence interval	Average daily bed error	Sensitivity	Specificity	Brier score	Expected calibration error
2018 1st Quarter	0.870468	0.864342	0.875981	0.821862	0.815898	0.827827	0.509851	0.614246	0.927623	0.124328	0.016912
2018 2nd Quarter	0.878449	0.872589	0.88375	0.833439	0.827629	0.839249	1.202293	0.612928	0.944503	0.119021	0.01533
2018 3rd Quarter	0.884498	0.879032	0.890365	0.842707	0.837097	0.848317	1.027719	0.62226	0.949853	0.114122	0.007713
2018 4th Quarter	0.883806	0.877979	0.8903	0.843617	0.838104	0.84913	5.581902	0.642047	0.930496	0.111403	0.030476
2019 1st Quarter	0.888781	0.882686	0.893568	0.852388	0.847009	0.857767	2.5198	0.629169	0.949566	0.107583	0.015462
2019 2nd Quarter	0.883425	0.878276	0.889564	0.855246	0.849949	0.860544	2.92562	0.613678	0.952637	0.106909	0.017532
2019 3rd Quarter	0.880467	0.874444	0.886946	0.853982	0.848622	0.859341	5.064206	0.608913	0.947334	0.10604	0.027635
2019 4th Quarter	0.885098	0.879167	0.890839	0.844485	0.839098	0.849873	3.31873	0.618584	0.944615	0.110524	0.018778
2020 1st Quarter	0.895973	0.890784	0.901225	0.856256	0.850993	0.861518	3.72264	0.62861	0.953883	0.103359	0.020342
2020 2nd Quarter	0.892488	0.886347	0.897878	0.851444	0.845678	0.85721	1.984686	0.64678	0.95345	0.107821	0.015869
2020 3rd Quarter	0.896991	0.891553	0.902558	0.859651	0.854381	0.864921	1.681576	0.620132	0.96615	0.102478	0.00991
2020 4th Quarter	0.899458	0.892929	0.904644	0.865394	0.860145	0.870643	4.022751	0.636173	0.960918	0.098859	0.022541
2021 1st Quarter	0.895623	0.889788	0.90072	0.860429	0.855	0.865859	3.10894	0.618599	0.963209	0.101454	0.018729
2021 2nd Quarter	0.891534	0.886119	0.897594	0.851153	0.845554	0.856752	4.004969	0.62586	0.951822	0.106635	0.023651
2021 3rd Quarter	0.895804	0.889432	0.901255	0.863794	0.858248	0.869341	3.204611	0.652685	0.965232	0.102033	0.020416
2021 4th Quarter	0.900395	0.894336	0.905822	0.865482	0.860126	0.870838	3.032189	0.649413	0.9682	0.099606	0.018661
2022 1st Quarter	0.885487	0.878965	0.891956	0.866	0.860187	0.871812	5.188739	0.648324	0.960635	0.102398	0.036301
2022 2nd Quarter	0.898509	0.892629	0.905027	0.873969	0.868483	0.879455	6.050347	0.667458	0.962234	0.096265	0.040299
2022 3rd Quarter	0.899718	0.893366	0.905417	0.87506	0.869695	0.880425	5.791981	0.663873	0.963186	0.094486	0.036721
2022 4th Quarter	0.898323	0.892907	0.90394	0.876326	0.871302	0.88135	7.839495	0.672014	0.958663	0.097554	0.043861
2023 1st Quarter	0.907999	0.901872	0.913344	0.882397	0.877184	0.88761	6.729675	0.69891	0.961126	0.094125	0.042278
2023 2nd Quarter	0.903384	0.89721	0.908918	0.87021	0.864687	0.875733	6.142541	0.69331	0.952857	0.101979	0.039601
2023 3rd Quarter	0.90734	0.901785	0.912713	0.870585	0.865056	0.876114	5.576982	0.704653	0.951545	0.101598	0.035935
2023 4th Quarter	0.902006	0.89588	0.907197	0.872377	0.866852	0.877902	6.051243	0.700644	0.953718	0.100892	0.039984

Quarterly retraining (quarterly model):

Quarter	Area Under the Receiver Operating Characteristic	Lower 95% confidence interval	Upper 95% confidence interval	Accuracy	Lower 95% confidence interval	Upper 95% confidence interval	Average daily bed error	Sensitivity	Specificity	Brier score	Expected calibration error
2018 1st Quarter	0.870468	0.865331	0.876114	0.821862	0.815898	0.827827	0.509851	0.614246	0.927623	0.124328	0.016912
2018 2nd Quarter	0.8783	0.87202	0.884559	0.834578	0.828784	0.840373	2.02172	0.595918	0.954783	0.118571	0.01371
2018 3rd Quarter	0.883373	0.878028	0.889155	0.841533	0.835906	0.84716	0.97799	0.623205	0.947649	0.114697	0.009724
2018 4th Quarter	0.882908	0.876568	0.888667	0.841158	0.835611	0.846706	6.291649	0.64563	0.925433	0.112975	0.034331
2019 1st Quarter	0.888061	0.882305	0.893213	0.850114	0.844701	0.855527	2.774253	0.630353	0.945786	0.108011	0.01561
2019 2nd Quarter	0.883355	0.877048	0.889611	0.85501	0.849709	0.860311	3.281497	0.614911	0.951809	0.106985	0.018149
2019 3rd Quarter	0.878403	0.872556	0.88521	0.853022	0.847648	0.858396	5.284727	0.613696	0.944187	0.107056	0.030045
2019 4th Quarter	0.884612	0.879504	0.890326	0.846959	0.841607	0.852312	3.230683	0.62589	0.944947	0.11047	0.017019
2020 1st Quarter	0.893133	0.887866	0.898582	0.854967	0.849685	0.860249	4.076687	0.634856	0.949364	0.104762	0.021977
2020 2nd Quarter	0.889799	0.88391	0.896448	0.848707	0.842897	0.854516	1.904501	0.649455	0.948016	0.109192	0.017622
2020 3rd Quarter	0.896177	0.890659	0.901724	0.854917	0.849573	0.860261	2.765571	0.635125	0.952645	0.103414	0.0156
2020 4th Quarter	0.896376	0.890343	0.901765	0.864655	0.859394	0.869917	3.870713	0.634708	0.960482	0.099749	0.021689
2021 1st Quarter	0.895618	0.889796	0.901803	0.857681	0.852207	0.863156	3.93882	0.633169	0.953101	0.102193	0.023335
2021 2nd Quarter	0.889927	0.883638	0.895529	0.850831	0.845227	0.856435	4.181705	0.629197	0.949865	0.107398	0.02451
2021 3rd Quarter	0.883055	0.87656	0.889299	0.845143	0.839293	0.850993	3.439791	0.624371	0.951224	0.111761	0.021428
2021 4th Quarter	0.900347	0.89455	0.905272	0.867535	0.862213	0.872856	4.277043	0.660761	0.965834	0.099597	0.025271
2022 1st Quarter	0.888766	0.88218	0.89494	0.869789	0.864047	0.875532	5.598075	0.658829	0.961505	0.100632	0.039194
2022 2nd Quarter	0.901591	0.895099	0.907108	0.875605	0.870149	0.88106	6.149693	0.672209	0.962538	0.094797	0.040745
2022 3rd Quarter	0.901731	0.895881	0.907489	0.879378	0.874093	0.884662	6.615814	0.676676	0.963963	0.093625	0.041595
2022 4th Quarter	0.899012	0.892655	0.904639	0.877721	0.872721	0.88272	8.216877	0.67539	0.959258	0.096725	0.045939
2023 1st Quarter	0.902571	0.896309	0.908269	0.878034	0.872738	0.88333	7.541091	0.691417	0.958106	0.098085	0.046785
2023 2nd Quarter	0.898084	0.892482	0.904123	0.863672	0.858032	0.869311	6.567748	0.680945	0.949041	0.106226	0.042021
2023 3rd Quarter	0.909423	0.903804	0.914906	0.872775	0.867286	0.878264	6.938418	0.717363	0.948602	0.100713	0.044603
2023 4th Quarter	0.904759	0.898444	0.909927	0.873804	0.868306	0.879303	7.000402	0.709527	0.951615	0.099537	0.045656

Supplementary Table 1-3: Full model results for each model in each quarter.

	XGBoost	Neural Network	Logistic Regression
Area Under the Receiver Operating Characteristic	0.861	0.841	0.816
Accuracy	0.815	0.801	0.801
Average daily bed error	0.034	4.17	0.12

Supplementary Table 4: Comparison of model types on the initial 2016 and 2017 training set for initial model selection.

	Handled as admissions (used for simulation)	Handled as discharges	Excluded from dataset

Area Under the Receiver Operating Characteristic	0.860	0.870	0.877
Accuracy	0.815	0.837	0.832
Average daily bed error	0.035	0.272	0.315

Supplementary Table 5: Sensitivity analysis comparing the results of different methods of handling the outcome disposition of a patient transfer in the initial 2016 and 2017 training set. Comparison was made between handling a transfer as an admission, discharge or exclusion from the dataset.

Metric	Difference-in-differences estimate	95% confidence interval
Area Under the Receiver Operating Characteristic	0.0009	−0.0017 to 0.0034
Accuracy	0.0019	−0.0034 to 0.0073
Brier score	−0.0009	−0.0030 to 0.0012
Expected Calibration Error	−0.0015	−0.0033 to 0.0002
Average daily bed error	−0.12	−0.71 to 0.46

Supplementary Table 6: Negative-control difference-in-differences results between the quarterly retraining model and the half-yearly retrained model

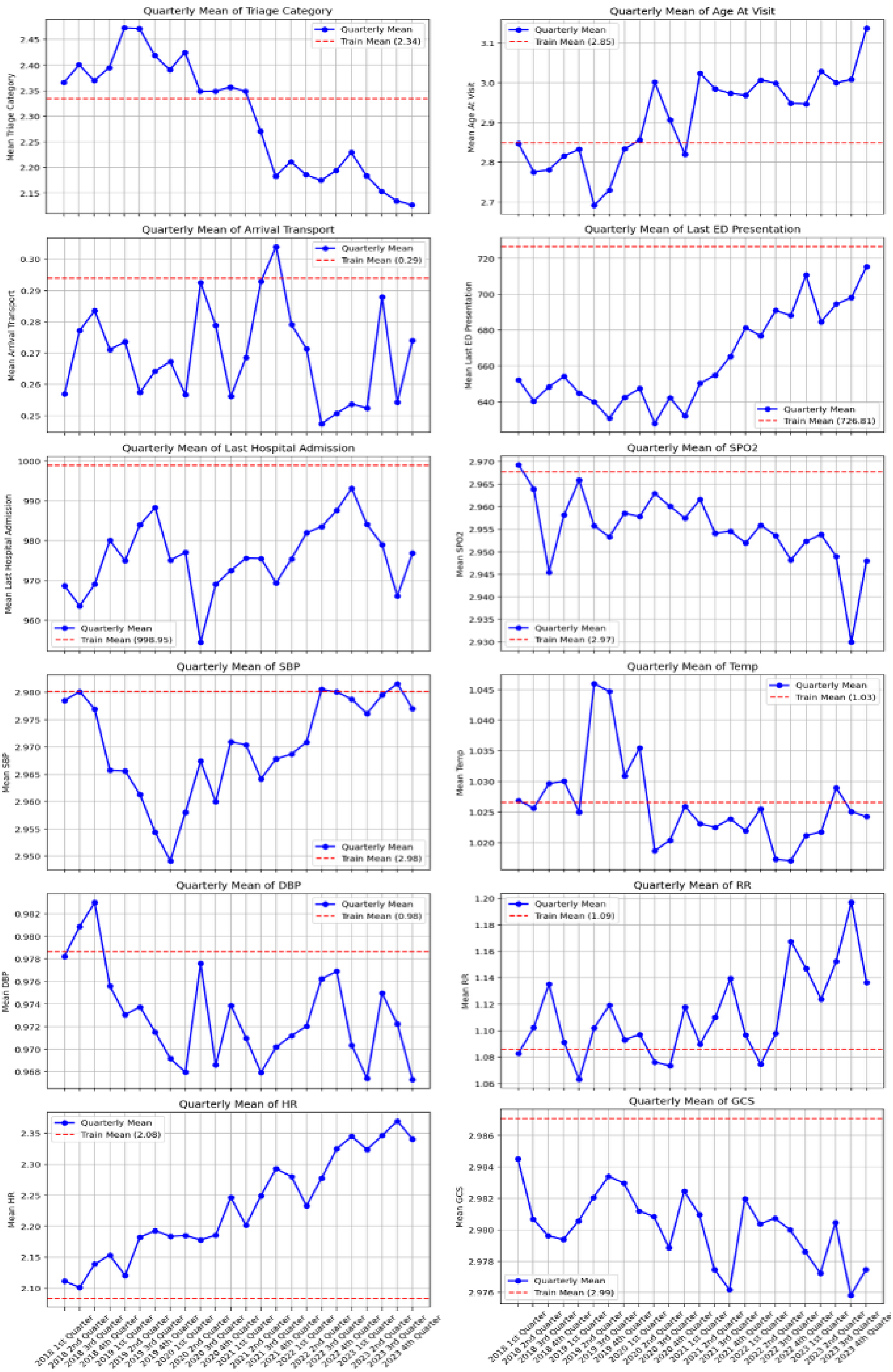
Quarterly Mean of Triage Category
Mean Triage Category
Quarterly Mean
Train Mean (2.34)
Quarterly Mean of Age At Visit
Mean Age At Visit
Quarterly Mean
Train Mean (2.85)
Quarterly Mean of Arrival Transport
Mean Arrival Transport
Quarterly Mean
Train Mean (0.29)
Quarterly Mean of Last ED Presentation
Mean Last ED Presentation
Quarterly Mean
Train Mean (726.81)
Quarterly Mean of Last Hospital Admission
Mean Last Hospital Admission
Quarterly Mean
Train Mean (998.95)
Quarterly Mean of SPO2
Mean SPO2
Quarterly Mean
Train Mean (2.97)
Quarterly Mean of SBP
Mean SBP
Quarterly Mean
Train Mean (2.98)
Quarterly Mean of Temp
Mean Temp
Quarterly Mean
Train Mean (1.03)
Quarterly Mean of DBP
Mean DBP
Quarterly Mean
Train Mean (0.98)
Quarterly Mean of RR
Mean RR
Quarterly Mean
Train Mean (1.09)
Quarterly Mean of HR
Mean HR
Quarterly Mean
Train Mean (2.08)
Quarterly Mean of GCS
Mean GCS
Quarterly Mean
Train Mean (2.99)
2018 1st Quarter
2018 2nd Quarter
2018 3rd Quarter
2018 4th Quarter
2019 1st Quarter
2019 2nd Quarter
2019 3rd Quarter
2019 4th Quarter
2020 1st Quarter
2020 2nd Quarter
2020 3rd Quarter
2020 4th Quarter
2021 1st Quarter
2021 2nd Quarter
2021 3rd Quarter
2021 4th Quarter
2022 1st Quarter
2022 2nd Quarter
2022 3rd Quarter
2022 4th Quarter
2023 1st Quarter
2023 2nd Quarter
2023 3rd Quarter
2023 4th Quarter

Supplementary Figure 2: Distribution of means for the top 10 variables over time across the simulation.

Key (categories defined by local vital sign charts):

Feature	Model category	Category definition
Triage Category	0	Priority 1
	1	Priority 2
	2	Priority 3
	3	Priority 4
	4	Priority 5
Arrival Mode	0	Private car/vehicle
	1	Ambulance Para
	2	Police Vehicle
	3	Other
	4	Ambulance - air
	5	Non-emerg pt transpt
	6	Comm/Pub Transport
	7	Helicopter
Temp [temp, T, t, Temp, TEMP]	0	<35 = hypothermia
	1	35-37.9 = normothermia/afebrile
	2	> 38 = febrile
Heart Rate (HR) [HR, hr, Hr]	0	<40 = severe bradycardia
	1	40-49 = bradycardia
	2	50-99 = normal heart rate
	3	100-119 = mild tachycardia
	4	120-139 = moderate tachycardia
	5	>/= 140 = severe tachycardia
Blood Pressure (SBP)	0	<75 = severe hypotension
	1	75-89 = moderate hypotension
	2	90 - 104 = mild hypotension
	3	105-169 = normotensive
	4	170-199 = hypertension
	5	>/= 200 = severe hypertension
Respiratory Rate (RR)	0	</=8 = hypoventilation
	1	9-20 = normal respiratory rate
	2	21-25 = mild tachypnoea
	3	26-30 = moderate tachypnoea
	4	31-35 = severe tachypnoea
	5	>/= 36 = very severe tachypnoea
Oxygen Saturation [SPO2, Spo2, spo2, Sat, SAT, sat, sats]	0	</= 84 = severe hypoxia
	1	85-89 = moderate hypoxia
	2	90-94 = mild hypoxia
	3	>/=95 = normal saturations

GCS	0<br>1<br>2<br>3	</= 8 = severely reduced conscious state<br>9-11 = moderately reduced conscious state<br>12-14 = mildly reduced conscious state<br>15 = normal conscious status
Departure Status	1<br><br><br>####<br><br><br><br>0	Admit - ED SSU<br>Admit this Hospital<br>Transferred<br>##<br>Did Not Wait<br>Discharge to RCF<br>Discharged Home<br>Escorted from Dept<br>Left at Own Risk<br>Disc AECU OP
Age At Visit	0	0-14
	1	15 - 25
	2	26- 39
	3	40 - 49
	4	50 - 59
	5	60 - 70
	6	71 - 80
	7	81- 100

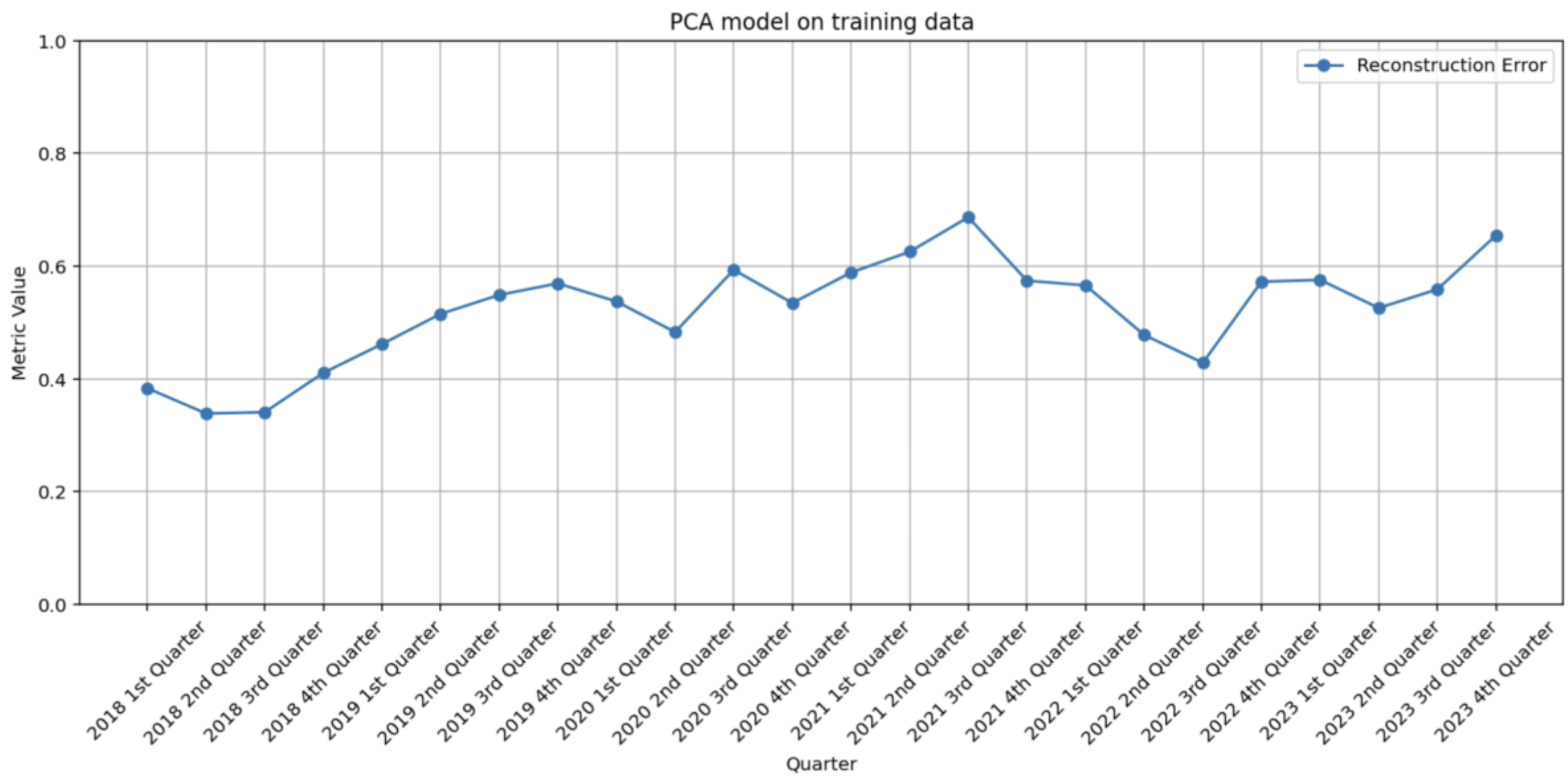

Supplementary Figure 3: PCA reconstruction error

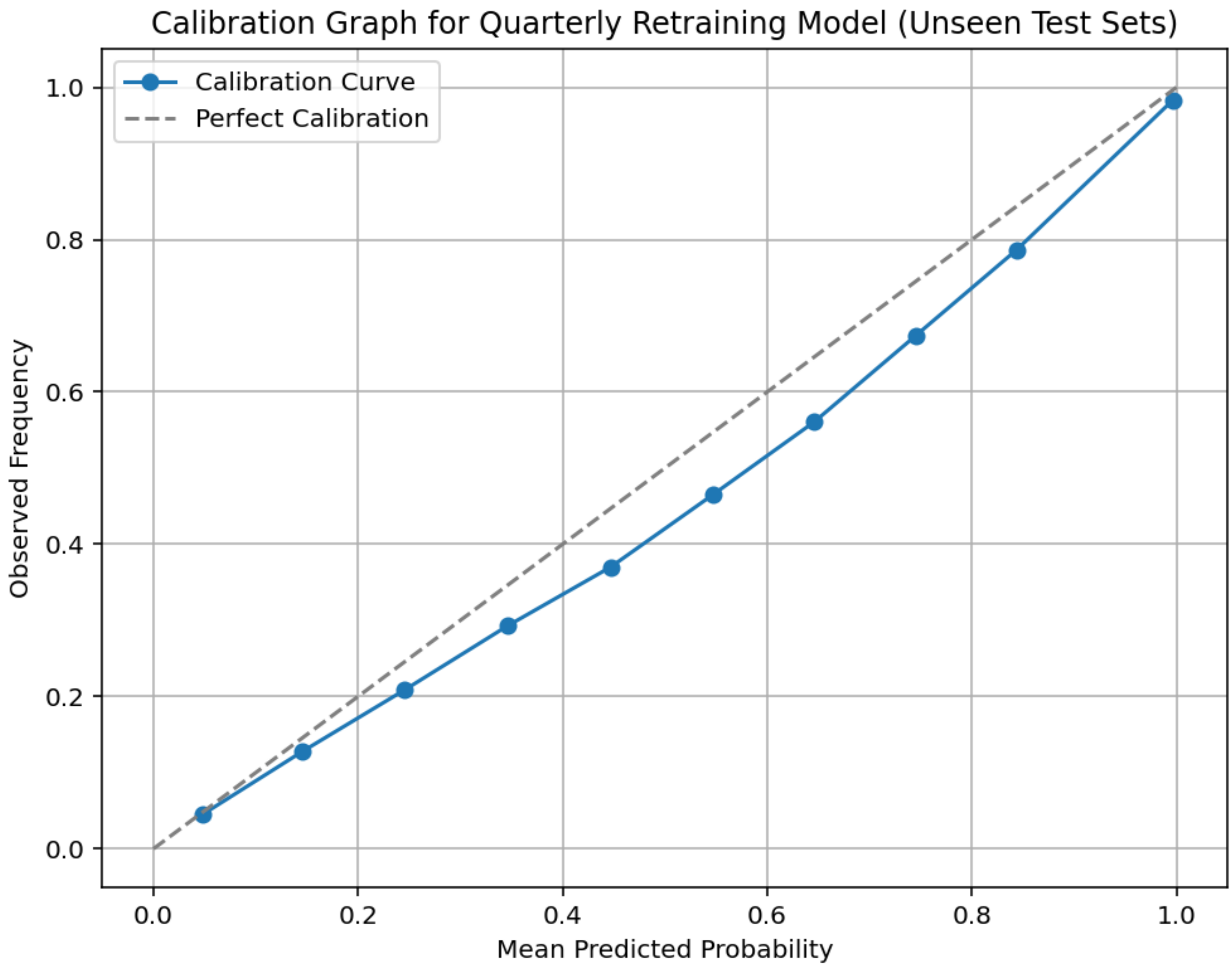

Supplementary Figure 4: Calibration graph for the quarterly model across the testing window.

Presenting Complaint Desc
Pain Abdo
Chest Pain
Other
Resp SOB
Short of Breath
Fever
Pain Limb
Generally Unwell
Injury Limb

Fall Mechanical
Pain Back
Vomiting/Diarrhoea
Wound Infection
CVA/TIA
Pain Chest
Psychiatric Disorder
Collapse
Suicidal
Dizziness/Vertigo
Cough
Nausea Vomiting
Multiple Problems
Fall Mechanism Unk
Headache
Cellulitis
Overdose/Poisoning
Pain Loin Flank
Urinary Symptoms
Injury Multi
Seizure
Arrhythmia Tachy
Altered Mental State
Injury Head
Altered Consc State
Confusion
Flu like symptoms
Diarrhoea
Arrhythmia
PR Bleed
Pregnancy Rel Issues
Neurological Deficit
Resp Wheeze
Sore Throat
Post-op complication
Urological Symptoms
Lethargy
Allergy
PV Bleed
Injury Laceration
Anaphylaxis
Endocrine
Pain Testicular

Rash
Haematuria
Injury Bite
Injury Facial
Epistaxis
Pain Headache
Foreign Body
Gynae
Haemetemesis
Malena
Arrhythmia Brady
Vomiting
URTI Symptoms
Pain Dental
Pain Eye
Weakness Unilateral
Injury Chest
Unsettled Child
Pain Ear
Anxiety
Visual disturbance
Haemoptysis
Irritable Child/Baby
Injury Eye
Injury Burn
Injury Bite Insect
Resp Stridor
Deliberate Self Harm
Convulsion Febrile
Pain Neck
Ingestion FB
Cardiac Arrest
Injury Nil
Multi Trauma
Chemical exposure
Injury Sexual
Renal colic
Medication/Script
Insect Bite / Sting
Plaster Change/Check
IVABs Administration
Appendicitis

Supplementary Table 7: Presenting complaint classifications.
